# Novel Delivery Systems for Checkpoint Inhibitors

**DOI:** 10.3390/medicines6030074

**Published:** 2019-07-11

**Authors:** Purushottam Lamichhane, Rahul Deshmukh, Julie A. Brown, Silvia Jakubski, Priyanka Parajuli, Todd Nolan, Dewan Raja, Mary Badawy, Thomas Yoon, Mark Zmiyiwsky, Narottam Lamichhane

**Affiliations:** 1 School of Dental Medicine, Lake Erie College of Osteopathic Medicine, 4800 Lakewood Ranch Blvd, Bradenton, FL 34211, USA; 2 School of Pharmacy, Lake Erie College of Osteopathic Medicine, 5000 Lakewood Ranch Blvd, Bradenton, FL 34211, USA; 3 Department of Biostatistics, University of Florida, Gainesville, FL 32611, USA; 4 Department of Internal Medicine, Southern Illinois University, Springfield, IL 62702, USA; 5 Department of Radiation Oncology, University of Maryland School of Medicine, Baltimore, MD 21201, USA

**Keywords:** immune-related adverse events, DNA-encoded monoclonal antibodies, platelets as delivery vehicles, resistance to checkpoint blockade, nanobodies, viral delivery of checkpoint inhibitors, hematopoietic stem cells, drug delivery systems, hydrogels, bacterial delivery

## Abstract

Checkpoint inhibition (CPI) therapies have been proven to be powerful clinical tools in treating cancers. FDA approvals and ongoing clinical development of checkpoint inhibitors for treatment of various cancers highlight the immense potential of checkpoint inhibitors as anti-cancer therapeutics. The occurrence of immune-related adverse events, however, is a major hindrance to the efficacy and use of checkpoint inhibitors as systemic therapies in a wide range of patients. Hence, methods of sustained and tumor-targeted delivery of checkpoint inhibitors are likely to improve efficacy while also decreasing toxic side effects. In this review, we summarize the findings of the studies that evaluated methods of tumor-targeted delivery of checkpoint inhibitors, review their strengths and weaknesses, and discuss the outlook for therapeutic use of these delivery methods.

## 1. Introduction

Over the last decade, cancer immunotherapy has emerged as an attractive addition and alternative to conventional treatment strategies, such as surgery, chemotherapy, and radiation therapy. While the utility of these conventional therapies remains indispensable, immunotherapy has added an extra dimension to the treatment regimens as a first line or subsequent therapy. Cancer immunotherapy aims to activate the body’s own immune system, such that effector immune cells are able to recognize and mount effective responses against cancer malignancies. Types of immunotherapies include checkpoint inhibitors (CPIs), co-stimulatory receptor agonists, adoptive cell transfer (ACT), cancer vaccines, cytokines, monoclonal antibodies, and oncolytic virus therapy [1,2,3,4]. Among these, checkpoint inhibition therapies have been the highlight of immunotherapy studies in the last decade. Immune checkpoint molecules include, but are not limited to, programmed death receptor 1 (PD-1), programmed death-ligand 1 (PD-L1), T-cell immunoglobulin and mucin domain-containing-3 (TIM-3), cytotoxic T-lymphocyte-associated protein 4 (CTLA-4), and lymphocyte-activation gene 3 (LAG-3) [5]. These checkpoint molecules are expressed on and are inducible on tumor cells and immune cells [6,7]. Receptor–ligand interactions of these checkpoint molecules lead to series of signaling cascades that result in suppression of immune responses against tumors, which can be rescued upon blockade of these molecules [8,9,10,11,12]. 

Successful preclinical studies and subsequent clinical trials have led to FDA (Food and Drug Administration) approvals of various checkpoint inhibitors for treatments of melanoma, pancreatic ductal adenocarcinoma, renal cell carcinoma, lymphoma, classical Hodgkin lymphoma, urothelial carcinoma, cervical cancer, non-small-cell lung cancer, triple negative breast cancer, and head and neck squamous cell carcinoma [13,14,15,16]. Despite impressive response rates in subsets of patients, checkpoint inhibition remains ineffective in many patients and immune-related adverse events limit its utility in a wide range of patients [6,17]. Commonly observed treatment-related adverse events associated with some checkpoint inhibitors and their incidences are summarized in Table 1. While the precise mechanisms of checkpoint inhibition-associated adverse events are unknown, T-cell activation, cytokine increase, and antibodies are likely to be some of the contributors [17,18]. Occurrence of fatigue and adverse events involving skin (rash, pruritus, and vitiligo), endocrine glands (hypothyroidism and hyperthyroidism), gastrointestinal tract (diarrhea and colitis), and liver (hepatitis, AST increase, and ALT increase) are common (Table 1). Other adverse events involving nervous, ocular, and cardiovascular systems are relatively rare [17,18,19]. When occurrences of adverse events of any grade are considered, the incidence has been found to be as high as 96% (Table 1). More importantly, grade III and IV adverse events are also common [19]. For example, 46% grade III and IV adverse events and 42% treatment discontinuation, associated with adverse events, have been reported for Ipilimumab (anti-CTLA-4; Bristol-Myers Squibb) treatment [20]. Furthermore, the incidence of adverse events tends to increase when a combination of checkpoint inhibitors is used for treatments [21,22]. In order to overcome these challenges, different tumor-targeted delivery vehicles are under development, which include, but are not limited to, delivery by nanoparticle and liposomes, viral vectors, platelets or hematopoietic stem cells, DNA encoded monoclonal antibodies, bacteria, injectable hydrogels, and matrix-binding checkpoint inhibitor conjugates. Nanoparticle and liposomal delivery of checkpoint inhibitors have been extensively discussed elsewhere [7,23,24]. In this review, we discuss some of the recent advances, as well as the challenges facing the novel delivery systems for checkpoint inhibition therapies. It is essential to note that delivery is not the only primary hindrance to the efficacy and utility of checkpoint inhibitor therapies. Various innate and adaptive resistance mechanisms have been shown to contribute to resistance to checkpoint inhibition therapies [6,25,26,27]. Tumor mutational load, defects in DNA-repair machinery, composition of gut microbiota, and presence or absence of tumor-infiltrating T cells have been shown to be some of the determinants of efficacy of checkpoint inhibition therapies [7,26,27,28,29,30,31,32]. Additionally, high cost associated with checkpoint inhibitor therapies represents yet another hindrance [33].

## 2. Delivery of Checkpoint Inhibitors by Platelets and Hematopoietic Stem Cells

Platelets are known to accumulate in surgical sites and also interact with circulating tumor cells [83]. These characteristics make them attractive delivery vehicles for tumor-targeted delivery of chemo- and immunotherapies [84,85,86]. Platelets have been studied as vehicles for delivery of PD-L1 blocking antibodies to surgical beds and circulating tumor cells [83]. Wang et al. found that post-surgery intravenous administration of platelets, conjugated to PD-L1 blocking antibodies on the surface via a bifunctional maleimide linker, can reduce recurrence and metastasis, and improve survival in the murine models of breast cancer and melanoma [83]. The release of conjugated PD-L1 antibody with the platelet-derived microparticles occurs upon platelet activation. In vivo, the circulation half-life of PD-L1 was significantly longer when administered with conjugated platelets. The circulation half-life of anti-PD-L1 upon platelet-conjugated anti-PD-L1 administration was 34.8 h compared to 5.2 h for free anti-PD-L1 administration [83]. Tumor recurrence was inhibited in 6 out of 8 mice receiving PD-L1 conjugated platelets (P-aPD-L1), while no inhibition was seen in the PBS (phosphate-buffered saline), platelets alone, or anti-PD-L1 alone groups [83]. Similarly, survival in P-aPD-L1 group was 75% after 60 days, while no mice survived past 30 days in other groups [83]. These results suggest that platelet-mediated delivery of checkpoint inhibitors to surgical beds post-surgical removal of tumors might be an important step toward preventing recurrence and metastases.

Additionally, the efficacy of anti-PD-1 decorated platelets conjugated to hematopoietic stem cells (S-P-aPD-1) has been tested against the murine model of acute myeloid leukemia (C1498) [87]. The hematopoietic stem cells (HSCs) were decorated with N-azidoacetylgalactosamine-tetraacylated (Ac_4_GalNAz) and platelets with dibenzocyclooctyne-PEG_4_-N-hydroxysuccinimidyl ester (BDCO-PEG_4_-NHS ester), and the two cells were incubated together to conjugate through a click reaction [87]. Intravenous administration of S-P-aPD-1 led to preferential homing to bone marrow (25-fold increase compared to control or platelets conjugated to anti-PD1) and local release of anti-PD1 post platelet activation, resulting in increased anti-leukemia response and improved survival [87]. Leukemia-bearing mice treated with S-P-aPD-1 had a survival rate of 87.5% at 80 days, while no mice survived past 40 days in control and single arms [87]. Additionally, treatment with S-P-aPD-1 resulted in resistance to re-challenge with leukemia cells. These results show that harnessing the homing capacity of HSCs and in situ activation potential of platelets to deliver checkpoint inhibitors may significantly improve their therapeutic efficacy against acute myeloid leukemia (AML). 

## 3. Delivery of Checkpoint Inhibitors by Viral Vectors

Delivery of checkpoint inhibitors via viral vectors represents another attractive modality. The ability to modify the viral surface with tumor-targeting moieties combined with the capability to stably deliver coding sequences for checkpoint inhibitors are immense advantages of viral vectors as therapeutic delivery vehicles. Additionally, viral vectors can be designed with deletions or additions of sequences to confer selective replication in tumor cells. Moreover, virus-induced cell lysis or immune activation boost the immune cells’ recruitment, activation, and anti-tumor responses. Because of the overall immune activating potential of tumor-targeted viruses, they are known to act as “in situ vaccines” [88,89,90]. Viral vector-induced immune activation can provide a substrate for checkpoint blockade to act upon and further stimulate anti-tumor immune responses. Immune responses to viral vectors, however, may also limit the efficacy of these delivery systems [91]. Dosing schedules, routes of administration, capsid modifications, and choice of specific-cell-restricted promoters may help overcome the barriers of immune responses against the viral vectors [91,92]. Below, we briefly summarize the results from various studies that have evaluated various viral vectors as checkpoint inhibitor delivery vehicles and discuss the challenges that need to be overcome prior to successful clinical development.

### 3.1. Retroviral Vectors

Retroviral delivery of PD-L1-targeted small hairpin RNA (shRNA) to tumor cells have been evaluated [93]. Treatment of PD-L1 expressing cancer cell lines with retroviral replication vectors (RRV) that express microRNA30-derived shRNA against PD-L1 (RRV-miRPDL1) resulted in sustained downregulation of PD-L1 expression and inhibited CD8^+^ T cell suppression [93]. The measured in vitro CD8^+^ T cell activation with RRV-miRPDL1, in trans-suppression lymphocyte assay, was similar to antibody-mediated PD-L1 blockade [93]. The RRV-miRPDL1 was not evaluated in vivo in this study and it remains to be seen if targeted delivery of RRV-miRPDL1 to tumors may result in improved efficacy and reduced toxicities compared to systemic therapies with PD-L1 blocking antibodies. There are indications of anti-tumor efficacy, however, with RRV encoding secreted single chain variable fragment (scFv) against PD-L1 (RRV-scFc-PDL1) in a murine model of orthotopic glioma [94]. Upon intracranial injection of RRV-scFc-PDL1 in glioma-bearing mice, approximately 50–150-fold less scFv-PD-L1 was detected in serum and survival was significantly improved (*p* = 0.0045) compared to systemic administration of PD-1 blocking antibody [94]. CPI therapy has failed to improve survival in patients of glioblastoma [95] and there is a lack of available studies indicating the benefit of CPI therapies in patients with glioblastoma [96]. CheckMate 143 (NCT 02017717), a phase III trial of Nivolumab (anti-PD-1) versus Bevacizumab (anti-VEGF-A) in patients of recurrent glioblastoma, reported that twelve-month overall survival (OS) for both treatments was 42%, with median OS in Nivolumab group at 9.8 months and in Bevacizumab group at 10 months [95,97]. Hence, the results of above-mentioned preclinical studies are significant and offer hope for successful clinical translation of CPI therapies in glioblastoma.

### 3.2. Adeno-associated Viral Vectors 

Tumor-targeted delivery of coding sequence of scFv-Fc fusion protein or full-length antibody against PD-1 using an adeno-associated virus (AAV) has also been evaluated [98]. Her2 receptor-targeted AAV (AAV capsid with Her2/neu-specific designed ankyrin repeat proteins (DARPins)) was packaged with the coding sequence for scFc-Fc fusion protein against PD-1 (Her2-AAV-PD1) [98]. While tumor-targeted delivery of Her2-AAV-PD1 in Her2/neu positive renal adenocarcinoma-bearing mice resulted in no significant difference in levels of anti-PD-1 in tumors compared to non-targeted delivery of scFc-Fc with AAV2 (1.9 ± 0.11 ng anti-PD-1/mg protein for Her2-AAV-PD−1 vs. 3.28 ± 1.22 ng anti-PD-1/mg protein for AAV2-PD−1), the anti-PD-1 levels were significantly decreased in liver and serum. The levels of anti-PD-1 in liver and serum were 5.12 ± 1.24 ng anti-PD-1/mg of protein and 1896 ± 378 ng/mL, respectively, for AAV2-PD-1 compared to 0.17 ± 0.01 ng anti-PD-1/mg of protein and 447.3 ± 36.7 ng/mL, respectively, for Her2-AAV-PD−1 [98]. Although the in vivo administration of Her2-AAV-PD-1 had only marginal anti-tumor activity and combination with cytostatic chemotherapy led to only modest improvement in tumor growth suppression [98], this targeted delivery method can be improved on for increased efficacy and decreased toxicities. To improve anti-tumor efficacy in future studies, it needs to be determined if the lower anti-tumor response in this study was due to the sub-optimal activity of the coded anti-PD-1, low levels of anti-PD-1, NK cell-mediated ADCC (antibody-dependent cellular cytoxicity) of T-cells, or the selected murine tumor model.

### 3.3. Oncolytic Viral Vectors

Many studies have shown the feasibility and improved efficacy of combining oncolytic virotherapy with systemic checkpoint blockade [99,100,101] or activation of costimulatory receptors [102]. Efficacy, mechanisms of actions of combined oncolytic virotherapy and checkpoint inhibition, and current clinical studies of this combination are discussed by various studies and publications [99,100,103,104]. Zamarin et al. showed that PD-L1 in the tumor microenvironment can mediate resistance to oncolytic virotherapy and that systemic blockade of PD-1/PD-L1 resulted in tumor rejection [105]. Various studies have evaluated the efficacy of tumor-targeted delivery of checkpoint inhibitors by oncolytic viruses [106,107,108]. Engeland et al. tested the efficacy of attenuated measles virus (MV) vectors, encoding for antibodies against PD-L1 (MVaPDL1) or CTLA-4 (MVaCTLA4), against murine model of melanoma (B16-CD20). Results show that intratumoral injections of MVaPDL1 only resulted in partial tumor regression in a subset of mice, but significantly improved survival compared to mock (*p* = 0.0016) or MV alone (*p* = 0.031) controls. In contrast, intratumoral injections of MVaCTLA4 decreased tumor burden compared to mock (*p* < 0.001) or MV alone (*p* < 0.05) controls in early time points (15 days), but failed to significantly improve overall survival [106]. When compared with the systemic checkpoint blockade, Engeland et al. found that there was no significant difference in survival between MVaPDL1 compared to MV plus systemic anti-PD-L1 treatment (*p* = 0.21). In contrast, MV plus systemic anti-CTLA-4 treatment resulted in significant improvement in survival compared to MVaCTLA4 (*p* = 0.0255) treatment [106]. Since systemic anti-PD-L1 treatment with MV resulted in survival times comparable to MVaPDL1 treatment and systemic anti-CTLA-4 treatment with MV resulted in significantly better survival times compared to MVaCTLA4, it remains to be seen if tumor-targeted delivery of checkpoint inhibitors by MV is superior to MV combination with systemic checkpoint blockade. Furthermore, no comparisons of toxicities were provided in this study. Authors propose that the superior efficacy of MV plus systemic anti-CTLA-4 treatment compared to MVaCTLA4 treatment may stem from the fact that CTLA-4 acts mainly in the lymphoid organs in the early phase of immune response, and hence systemic therapy with anti-CTLA-4 might be a better combination with MV treatment [106]. However, it is clear from these results that therapeutic benefit of different checkpoint inhibitors may vary depending on the mode of delivery (systemic versus tumor-targeted) when combined with MV treatment, and that further biological and mechanistic insights are necessary prior to making any generalized inferences about the efficacy and potential of this delivery method for checkpoint inhibitors.

Similarly, Du et al. showed that intratumoral treatment of subcutaneous lung cancer (CMT-64)-bearing mice with replication competent oncolytic adenovirus encoding for anti-CTLA-4 antibody resulted in decreased tumor burden, but data on toxicities were not available and no comparison with adenovirus plus systemic anti-CTLA-4 treatment was presented [108]. 

Kleinpeter et al. compared the antitumor efficacy of vectorized Western Reserve (WR) oncolytic vaccinia virus to that of wild type WR virus plus systemic PD-1 blockade (WR + anti-PD-1) in a murine model of fibrosarcoma (MCA205) [109]. The vectorized viruses encoded for whole antibody (WR-mAb), fragment antigen-binding (WR-Fab), or single-chain variable fragment (WR-scFv) against murine PD-1 [109]. Intratumoral treatment of WR-mAb significantly increased tumor/serum ratio (*p* < 0.05) of mAb compared to the intratumoral injection of anti-PD-1 antibody [109]. The survival and tumor size, however, were similar in both the WR-mAb treatment and WR+anti-PD-1 treatment groups and comparison of toxicities were not reported [109].

Bartee et al. evaluated the efficacy of intratumoral delivery of recombinant myxoma virus, encoding the soluble form of PD-1 (MYV-PD1) in the murine model of melanoma (B16/F10) [107]. Compared to the treatment combination of myxoma virus and systemic PD-1 blockade, MYV-PD1 resulted in significantly increased complete responses (*p* = 0.04) and survival (*p* = 0.01) with significantly lower toxicity (alopecia; *p* < 0.05) [107]. The anti-tumor efficacy of this treatment was dependent on CD8^+^ T cells. It is important to note that MYV-PD1 as monotherapy did not improve survival or decrease the number of metastatic lesions when tested in a metastatic model of melanoma, representing an important hurdle that needs to be addressed for this treatment to have a chance of effective translation in humans. The size and numbers of metastatic lesions, however, were significantly decreased (*p* < 0.001) and survival significantly improved (*p* < 0.001) when MYV-PD1 was combined with CD4 T cell depletion [107]. 

While delivery of checkpoint inhibitors by oncolytic viruses appears to be therapeutically beneficial and offers opportunities for tumor targeting, sustained in situ production of checkpoint inhibitors, and decreased costs, it is essential that further studies with appropriate controls and comparison of toxicities are performed to determine if there are significant differences in responses based on the types of viruses used. Likewise, toxicities and efficacies of targeted delivery of checkpoint inhibitors with oncolytic viruses must be compared to combination with systemic checkpoint inhibitor treatment for different checkpoint molecules to determine the superiority between the two methods. 

## 4. Delivery of Checkpoint Inhibitors as DNA-encoded Monoclonal Antibodies (DMAbs)

With the goal of improving efficacies and circumventing the issues of frequent dosing, high cost, and complex manufacturing processes for checkpoint inhibitors; the DNA-encoded monoclonal antibody (DMAb) technique has been developed for delivery of checkpoint inhibitors and evaluated for efficacy in preclinical studies [110,111]. Duperret et al. [110] showed that muscle injection and electroporation of a single dose of DMAbs encoding human anti-CTLA-4 (DMAb-hCTLA4) in mice results in sustained production of anti-CTLA-4 antibody (above 15 μg/mL) for over a year; similar to the steady-state serum concentration of ipilimumab at 21.8 μg/mL under the recommended regimen [111]. In murine models of fibrosarcoma (Sa1N) and colon carcinoma (CT26), injection of a single dose of DMAbs (100 μg) encoding murine anti-CTLA-4 resulted in complete responses and suppressed tumor growth similar to 3 doses of recombinant anti-CTLA-4 treatment at 10 ug/injection. Perales-Puchalt et al. extended this study to generate DMAbs encoding human anti-PD-1 [111]. The anti-PD-1 generated this way retained the PD-1 binding capacity similar to the recombinant anti-PD-1 antibody [111], but in vivo anti-tumor responses were not evaluated. Once again, these studies did not report on comparisons of toxicities. 

Incomplete or missing toxicity studies and a lack of incorporation of an “off switch” to shut off the prolonged antibody production, if necessary, are significant hurdles that need to be addressed prior to successful translation of DMAb technology for checkpoint inhibition in humans. Additionally, it is essential to realize that different doses and schedules influence the efficacies and related toxicities to checkpoint blockade therapies [112], and hence methods to control the expression of DMAbs may be required to harness the full potential of this checkpoint inhibitor delivery technology.

## 5. Delivery of Checkpoint Inhibitors by Bacteria

Nanobodies are single domain antibodies (~15 kDa) [113] derived from the heavy chain variable domain. Nanobodies retain their binding specificity, but compared to whole antibodies, Fab fragments, and single-chain variable fragments, they are more hydrophilic and have characteristics such as increased solubility, stability, and chemical resistance [114]. These make them attractive candidates for applications in diagnostics, imaging, and therapies, and are at various stages of development as anti-cancer agents [115]. With the recent approval of the first therapeutic nanobody (targeting von Willebrand factor) for treatment of acquired thrombotic thrombocytopenic purpura [115], there is much to look forward to in the successful application of nanobodies in cancer immunotherapies. While design and efficacy of nanobodies targeting checkpoint molecules have been evaluated [116,117,118], studies evaluating the feasibility and efficacy of targeted delivery of nanobodies are scarce. 

While bacteria have been explored as a delivery vehicle for various anti-cancer agents [119,120,121,122,123,124,125], their use as delivery vehicles for checkpoint inhibitors has been limited [126,127]. In a study by Gurbatri et al., the probiotic strain of *E. coli* (*E. coli* Nissle 1917) containing plasmid for single domain antibodies (nanobodies) against either PD-L1 or CTLA-4 was evaluated for efficacy in a murine model of colorectal cancer (CT26) [126]. Gurbatri et al. utilized one plasmid system such that a quorum sensing promoter drove transcription of both the quorum sensing genes and phage-derived lysis gene, creating a synchronized lysis circuit (SLC) [126]. This allowed for the release of anti-PD-L1 or anti-CTLA-4 nanobodies through bacterial lysis when the bacterial mass reached a critical density, resulting in delivery of a high dose of nanobodies. Although bacterial delivery of anti-PD-L1 nanobodies (SLC-PDL1) had similar efficacy as systemic PD-L1 blockade, there were more necrotic areas and neutrophil infiltration into the tumors with SLC-PDL1 treatment [126]. Similarly, the bacterial delivery of anti-CTLA-4 nanobodies (SLC-CTLA4) had efficacy comparable to systemic CTLA-4 blockade. The combination of SLC-PDL1 and SLC-CTLA4 led to significantly decreased tumor volumes, compared to each monotherapy, in both the CT26 and A20 tumor models, although comparison with the combination of systemic PD-L1 and CTLA-4 blockade was not performed [126]. The toxicities associated with systemic checkpoint blockade therapies, however, seemed to be significantly eliminated with this delivery method. In the 4T1 model of low immunogenic breast cancer, Gurbatri et al. showed that while systemic anti-PD-L1 antibody treatment led to severe toxicities and deaths, SLC-PDL1 treatment led to no significant toxicities [126]. Similarly, in the colon cancer model (CT26), toxicities observed in the SLC-CTLA4 group were less compared to the group with systemic CTLA-4 blockade (increase in body weight by ~30% compared to ~10%) [126]. Even with the combination of SLC-PDL1 and SLC-CTLA4, no significant toxicities were observed in either the CT26 or A20 model [126].

Bacterial delivery of small interfering RNA (siRNA) targeting PD-1 has also been evaluated [127]. In a murine model of melanoma (B16), Zhao et al. found that intraperitoneal injection of attenuated *Salmonella Typhimurium* carrying a plasmid for PD-1 siRNA (ST-siPD1) preferentially accumulated in tumors (*p* < 0.01 compared to other organs) [127]. Compared to mock treatment or treatment with bacteria containing a plasmid for scrambled siRNA, intratumoral injection of ST-siPD1 led to significantly improved survival (*p* < 0.01), decreased PD-1 protein expression (*p* < 0.05), and decreased tumor weight (*p* < 0.05) [127]. Comparison with systemic PD-1 blockade, however, was not performed. 

In both of the above-mentioned studies, treatments were delivered through intratumoral injections [126,127]. Since this method is not always feasible in patients and given that bacteria have been found to be present and preferentially grow in tumors [128], it will be important to determine if oral administration of bacteria could result in similar anti-tumor effects compared to intratumoral injections. There are indications elsewhere that oral administration of bacteria as delivery vehicles for chemotherapies can result in antitumor activity [119]. Thus, this method represents a significant step forward in delivering checkpoint inhibitors to the tumors in a safe manner. With recent works demonstrating the role of gut microbes in determining the response to checkpoint blockade therapies [31,32,129,130,131], this delivery method might serve a dual purpose with the correct choice of bacteria as a programmable delivery vehicle.

## 6. Delivery of Checkpoint Inhibitors by Matrix-binding Checkpoint Inhibitor Conjugates

Immune-related adverse events (IRAEs) are common with systemic checkpoint blockade therapies, and hence reduce their utility in many patients despite their remarkable therapeutic effects in subsets of patients [17]. Intratumoral or peritumoral delivery and prolonged retention of checkpoint inhibitors in the tumors may reduce the serum concentration of checkpoint inhibitors, reduce IRAEs, and improve efficacy [1,132]. Ishihara et al. tested the efficacy of peritumoral delivery of extracellular matrix-binding protein conjugated to anti-PD-L1 (PIGF2-PDL1) or anti-CTLA-4 (PIGF2-CTLA4) [132]. The matrix-binding protein was a peptide derived from placental growth factor 2 (PIGF2_123–144_), which showed high affinity for eight different extracellular matrix (ECM) proteins. Peritumoral delivery of PIGF2-PDL1 or PIGF2-CTLA4 led to increased tumor retention and decreased plasma concentration (*p* < 0.01) of these antibodies compared to intraperitoneal or peritumoral administration of unconjugated antibodies [132]. Compared to intratumoral or peritumoral treatment with a combination of anti-PD-L1 and anti-CTLA-4 antibodies, treatment with combined PIGF2-PDL1 and PIGF2 led to delayed tumor growth and improved survival in an implantable murine model of melanoma (*p* < 0.05) and in genetically engineered mouse models of melanoma (*p* < 0.05) and breast cancer (*p* < 0.05) [132]. Interestingly, this treatment also led to abscopal effects, suggesting the induction of systemic anti-tumor immunity. Additionally, it was observed that lower plasma levels of antibodies were present with PIGF2-PDL1 or PIGF2-CTLA4 treatment, and no mice (nonobese diabetic mice) developed diabetes with PIGF2-PDL1 treatment compared to 100% diabetes incidence in the unconjugated anti-PD-L1 treatment group [132]. These results suggest that peritumoral delivery of checkpoint inhibitors by the matrix-binding checkpoint inhibitor conjugates might lead to increased tumor retention of checkpoint inhibitors, circumvention of the toxicities associated with systemic checkpoint blockade, and improved survival. For inaccessible tumors, it will be important to determine if intraperitoneal administration of matrix-binding checkpoint inhibitor conjugates results in similar toxicity and efficacy profiles as peritumoral administration. 

## 7. Delivery of Checkpoint Inhibitors by Injectable Hydrogels.

Hydrogel-based delivery systems have been evaluated for therapeutic efficacy and toxicity of checkpoint inhibitors against various tumors [112,133,134,135]. Hydrogels are composed of cross-linked polymeric material networks that have the ability to swell, absorb water, and retain a significant amount of water within their structures [136]. Hydrogels can be designed for any shape or size and their cross-linked networks can prevent degradation of encapsulated drugs or bioactive materials from low pH or enzymes in vivo [137,138]. Additionally, hydrogels can be designed to be biodegradable, allow for easy encapsulation of drugs and bioactive materials, and their mesh size is changeable to fine-tune the controlled release of the payload [137,138]. All these features make hydrogels attractive delivery vehicles for local and sustained release of anti-cancer therapeutics. 

Wang et al. designed an injectable reactive oxygen species (ROS)-degradable hydrogel scaffold that encapsulated gemcitabine and an anti-PD-L1 antibody [134]. When injected into the murine model of low-immunogenic breast cancer (4T1), the ROS-induced degradation of the hydrogel first led to release of gemcitabine causing cancer cell death, creating an immunogenic phenotype in the tumor microenvironment [134]. The delayed release of anti-PD-L1, hence, was able to stimulate antitumor immunity, leading to significantly delayed tumor growth (< 0.01) and improved survival (*p* < 0.05) compared to treatments with a single agent (gemcitabine or anti-PD-L1 hydrogels) alone. Similar results were obtained when tested in a murine model of melanoma (B16F10) [134]. 

In another study, Li et al. used an alginate polymer-based hydrogel system to simultaneously deliver celecoxib (cyclooxygenase-2 inhibitor) and anti-PD-1 antibody to tumors via tumor-adjacent injection [133]. Combined delivery of celecoxib and anti-PD-1 resulted in a significant delay in tumor growth and improved survival in murine models of melanoma (B16F10) and metastatic breast cancer (4T1) compared to hydrogel delivery of celecoxib or anti-PD-1 alone [133]. These results were associated with increased tumor infiltration of IFNγ^+^CD4^+^ and IFNγ^+^ CD8^+^ T cells and decreased frequency of regulatory T cells (T_regs_) and myeloid-derived suppressor cells (MDSCs) [133]. In this study, both the celecoxib and anti-PD-1 were found to be present at high concentrations in tumor tissues, as well as in the serum for prolonged periods of time [133]. The risks of autoimmunity posed by sustained high levels of anti-PD-1 in serum may be circumvented by decreasing the dose delivered by hydrogels. In yet another study, Song et al. used PEG-b-poly(L-alanine) hydrogel to encapsulate and deliver tumor cell lysates, granulocyte–macrophage colony stimulating factor (GMCSF), anti-PD-1, and anti-CTLA-4 simultaneously [135]. In the murine model of melanoma (B16), the combined delivery led to significantly delayed tumor growth (*p* < 0.01) and exhibited no significant toxicity (body weight) compared to hydrogel delivery of vaccine (GMCSF + tumor cell lysate) alone or in combination with either anti-PD-1 or anti-CTLA-4, or compared to combined delivery of vaccine and anti-PD-1 plus anti-CTLA-4 in a solution [135]. Further toxicity studies showed no differences in blood urea nitrogen, decreased alanine aminotransferase, and increased platelets, leukocytes, and hemoglobin counts with hydrogel delivery compared to intraperitoneal delivery of the vaccine plus anti-PD-1 and anti-CTLA-4 in a solution [135]. These changes were not significant for the hydrogel delivery when compared with the control treatment [135]. These results demonstrate that hydrogel-based local delivery of checkpoint inhibitors in combination with vaccines or chemotherapies may improve therapeutic efficacy in various cancers without risk of systemic toxicities associated with systemic checkpoint inhibitor treatment. More importantly, this technology could be further tuned to control the release kinetics of each therapy to desired rates for sustained local delivery to the tumors for improved efficacy and decreased toxicities.

## 8. Translational Outlook

To the best of our knowledge, currently there are no reported trial results or ongoing clinical trials for the delivery of checkpoint inhibitors by the vehicles discussed in this manuscript. There are, however, ongoing and completed clinical trials evaluating the efficacy of some of these therapeutic vehicles in combination with systemic checkpoint blockade. For example, Talimogene laherparepvec (T-Vec; HSV-1 backbone), the first FDA approved oncolytic viral therapy against melanoma [99], was found to generate an objective response (OR) of 39% when combined with Ipilimumab compared to 18% OR with Ipilimumab alone in an open-label Phase II study (NCT01740297) in patients with advanced, unresectable melanoma [139]. Multiple ongoing clinical trials using the combination of oncolytic viruses and checkpoint inhibitors are underway [99]. Similarly, while platelets have been studied as drug delivery vehicles [85], no clinical trial is currently underway for evaluation of platelets as drug delivery vehicles. Likewise, while no clinical trials are evaluating hematopoietic stem cells as delivery vehicles for checkpoint inhibitors, trials are underway to evaluate the efficacy of checkpoint blockade in patients with AML and myelodysplastic syndrome who have received hematopoietic stem cell transplantation (NCT02846376) and in patients with multiple myeloma or T/B-cell lymphoma at risk of recurrence post stem cell transplantation (NCT02681302). Excitingly, clinical trials are underway to evaluate the efficacy of DMAbs as delivery vehicles (NCT03831503, NCT03439085). Recent FDA approval to start the first Phase I open-label trial (NCT03831503) of DMAbs encoding for antibodies against Zika virus in healthy volunteers and the planned clinical trial of PD-1 inhibitor-encoding DMAbs from Inovio Pharmaceuticals suggest that we may be closer to finding out the clinical efficacy of DMAbs as delivery vehicles. Clinical evaluations of bacteria as drug delivery vehicles are also underway (NCT03751007, NCT03234465, NCT03516487). For example, a Phase 1b/2a study (NCT03751007) aims to evaluate the safety and tolerability of *Lactococcus lactis* (modified to deliver human proinsulin and the cytokine IL-10) in recently diagnosed patients of Type 1 Diabetes Mellitus. While no current clinical trials have directly evaluated the safety and efficacy of the checkpoint inhibitors’ delivery by vehicles discussed in this manuscript, the completed trial results and the ongoing and planned clinical trials of these delivery vehicles in the near future should provide clues to their potential for successful clinical translation as delivery vehicles for checkpoint inhibitors.

## 9. Summary

The utility of systemic checkpoint blockade in a wide range of cancer patients is hindered by the low immunogenicity of some tumors, and more importantly due to immune-related adverse events that often develop with these treatments. Methods of tumor-targeted delivery coupled with sustained expression and release of checkpoint molecules allow for targeting these inhibitors to the desired cells. and hence improving efficacy and avoiding toxicities and off-target effects. Additionally, these delivery technologies can simultaneously deliver cytotoxic agents or vaccines with checkpoint inhibitors to enhance the immunogenicity of low immunogenic tumors, such that local checkpoint blockade can further enhance the anti-tumor immune responses. Tumor targeted delivery and sustained release and expression also allows for the combination of chemotherapeutics with checkpoint inhibition, which would otherwise be too toxic to deliver systemically, thus offering additional avenues of therapies for patients. Each delivery system, however, has its strengths and weaknesses (Table 2). Hence, the choice of delivery vehicles and potential to overcome associated weaknesses are paramount in successful targeted delivery of checkpoint inhibitors for different malignancies. While preclinical studies have shown immense promise, further studies should address the issues with the feasibility of administration routes, incorporation of an “off switch” for in vivo checkpoint inhibitor expression technologies, tunable kinetics of controlled release methods, and extensive comparison of toxicity profiles prior to successful clinical translation of these delivery methods.

## Figures and Tables

**Table 1:** Summary of treatment-related adverse events associated with select checkpoint inhibitor therapies.

Treatment-Related Adverse Events	PD-1 Inhibitor		PD-L1 Inhibitor	CTLA-4 Inhibitor
	Nivolumab	Pembrolizumab	Atezolizumab	Ipilimumab
Any adverse event	30–85%	40–75%	16–67%	55–96%
**Skin**				
Pruritus	2–22%	4–21%	5–14%	25–36%
Rash	4–24%	8–21%	5–15%	15–34%
Vitiligo	3–11%	9–25%	NR	2–9%
**Gastrointestinal**				
Diarrhea	7–22%	7–20%	5–20%	23–46%
Colitis	1–9%	1–4%	1–2%	7–25%
**Hepatic**				
ALT Increase	1–8%	2–8%	2–4%	0–15%
AST increase	1–12%	3–10%	2–4%	1–13%
Hepatitis	0–5%	1–2%	1–2%	0–9%
**Endocrine**				
Hypothyroidism	4–10%	7–14%	2–7%	1–15%
Hyperthyroidism	0–5%	3–10%	1%	0–2%
Hypophysitis	<1%	1–2%	<1%	2–16%
**Respiratory**				
Pneumonitis	1–9%	2–6%	1–4%	0.4–4%
**Source Publications**	[20,34,35,36,37,38,39,40,41,42,43,44,45,46,47,48,49]	[48,50,51,52,53,54,55,56,57,58,59,60,61,62,63,64,65]	[66,67,68,69,70,71,72,73,74,75]	[20,46,47,48,49,65,76,77,78,79,80,81,82]

ALT: Alanine aminotransferase; AST: Aspartate aminotransferase; NR: No Reported Data.

**Table 2:** Summary of strengths and weaknesses of delivery systems.

Delivery System	Strengths	Weaknesses	Source Publications
Platelets	Readily available and biocompatible Easily activated to release drugs Preferred homing to wounds/injury sites make platelets ideal candidate for post-surgical drug delivery to surgical sites High loading efficacy Easy surface modifications Encapsulated drugs are protected from physical stress and immune system Controlled release from platelets can be achieved by induction with agonists	Platelets can be easily deformed and aggregated Complex ex vivo processing for loading of therapeutics Agents used to prevent platelet aggregation can be harmful to human body Limitations with storage Unexpected activation and release of therapeutics may occur in unintended sites	[83,85,86,140,141,142,143,144]
DMAbs	Robust expression in vivo Transient expression Well tolerated and little risk of integration Inexpensive to produce and can be administered repeatedly Deemed safe in early clinical studies	Pain associated with site of electroporation Low efficiency in large animals/humans Restricted to protein therapeutics Induction of antibodies against DNA is possible	[110,145,146,147,148,149]
Viral Vectors	Stimulates immune system Can be easily genetically engineered for tumor targeting Can target both dividing and non-dividing cells Can be engineered for selective replication in target cells High levels of expression of the therapeutics for prolonged period Capacity for incorporating multiple genes simultaneously Potential for systemic delivery	Risk of restored virulence and seroconversion in vivo Anti-viral responses may limit efficacy and dosing Complex engineering process to avoid interference by pre-existing immunity High safety and regulatory standards Risks of random integration and oncogene activation	[90,150,151,152,153,154]
Extracellular Matrix Binding Protein	Efficient targeting of the whole TME (cancer cells and supporting cells) Prolonged tissue retention of the therapeutics	Selection of tumor-restricted ECM might be challenging Limited information on stability and efficacy for systemic administration	[132,155]
Bacteria	Preferred accumulation and proliferation in tumor tissues Ability to penetrate tissues Expression of chemotactic receptors for migration to TME Can be easily genetically engineered to carry various therapeutics and targeting moieties Modifiable promotors that respond to different agents (small molecules, radiation, etc.) Ability to stimulate immune system Potential for oral delivery	Residual bacterial virulence might be an issue in immunocompromised patients Effective colonization and targeting may not be achieved in small metastatic lesions Concerns of genetic instability, mutations, and horizontal gene transfers Existing immunity against the bacterial vectors may reduce efficacy	[120,123,128,156,157,158,159]
Hydrogels	Easy drug encapsulation and protection of therapeutics from degradation by enzymes, low pH, etc. Biocompatible and biodegradable Tunable shape and mesh size for controlled release of drugs Prolonged retention; localized and sustained drug release Low likelihood of systemic toxicity Low cost of preparation Minimally invasive (injectable) Potential for oral delivery	Issues with viscosity Poor mechanical stability Difficult to sterilize Issues of biocompatibility with synthetic hydrogels Limitations with encapsulation and delivery of hydrophobic drugs	[136,137,138,160,161,162,163,164]

DMAbs: DNA-encoded Monoclonal Antibodies; TME: Tumor microenvironment.

## Abbreviations

Programmed Death Receptor 1	PD-1
Programmed Death-Ligand 1	PD-L1
Cytotoxic T-lymphocyte-associated protein 4	CTLA-4
Checkpoint Inhibition	CPI
Tumor Microenvironment	TME
Alanine Aminotransferase	ALT
Aspartate Aminotransferase	AST
Treatment-Related Adverse Events	TRAEs
Immune-Related Adverse Events	iRAEs
Reactive Oxygen Species	ROS
Granulocyte–Macrophage Colony Stimulating Factor	GMCSF
Acute Myeloid Leukemia	AML
Adeno-Associated Virus	AAV
Retroviral Replication Vectors	RRV
Hematopoietic Stem Cells	HSCs
DNA-encoded Monoclonal Antibodies	DMAbs
